# Distinct deposition of amyloid-β species in brains with Alzheimer’s disease pathology visualized with MALDI imaging mass spectrometry

**DOI:** 10.1186/s40478-017-0477-x

**Published:** 2017-10-16

**Authors:** Nobuto Kakuda, Tomohiro Miyasaka, Noriyuki Iwasaki, Takashi Nirasawa, Satoko Wada-Kakuda, Junko Takahashi-Fujigasaki, Shigeo Murayama, Yasuo Ihara, Masaya Ikegawa

**Affiliations:** 1 Genomics, Proteomics and Biomedical Functions, Department of Life and Medical Systems, Faculty of Life and Medical Sciences, Doshisha University, Kyoto, Japan; 2 Neuropathology, Department of Life and Medical Systems, Faculty of Life and Medical Sciences, Doshisha University, Kyoto, Japan; 3 Bruker Daltonics K.K, Yokohama, Japan; 4 Neuropathology, The Brain Bank for Aging Research, Tokyo Metropolitan Geriatric Hospital and Institute of Gerontology, Tokyo, Japan; 5 Graduate School of Brain Science, Doshisha University, Kyoto, Japan

**Keywords:** Amyloid β, Alzheimer’s disease, Cerebral amyloid angiopathy, Imaging mass spectrometry, C- and N-terminal variations of Aβ, Senile plaques, γ-secretase, Perivascular space

## Abstract

Amyloid β (Aβ) deposition in the brain is an early and invariable feature of Alzheimer’s disease (AD). The Aβ peptides are composed of about 40 amino acids and are generated from amyloid precursor proteins (APP), by β- and γ-secretases. The distribution of individual Aβ peptides in the brains of aged people, and those suffering from AD and cerebral amyloid angiopathy (CAA), is not fully characterized. We employed the matrix-assisted laser desorption/ionization-imaging mass spectrometry (MALDI-IMS) to illustrate the spatial distribution of a broad range of Aβ species in human autopsied brains. With technical advancements such as formic acid pretreatment of frozen autopsied brain samples, we have: i) demonstrated that Aβ1–42 and Aβ1–43 were selectively deposited in senile plaques while full-length Aβ peptides such as Aβ1–36, 1–37, 1–38, 1–39, 1–40, and Aβ1–41 were deposited in leptomeningeal blood vessels. ii) Visualized distinct depositions of N-terminal truncated Aβ40 and Aβ42, including pyroglutamate modified at Glu-3 (N3pE), only with IMS for the first time. iii) Demonstrated that one single amino acid alteration at the C-terminus between Aβ1–42 and Aβ1–41 results in profound changes in their distribution pattern. In vitro*,* this can be attributed to the difference in the self-aggregation ability amongst Aβ1–40, Aβ1–41, and Aβ1–42. These observations were further confirmed with immunohistochemistry (IHC), using the newly developed anti-Aβ1–41 antibody. Here, distinct depositions of truncated and/or modified C- and N-terminal fragments of Aβs in AD and CAA brains with MALDI-IMS were visualized in a spacio-temporal specific manner. Specifically, Aβ1–41 was detected both with MALDI-IMS and IHC suggesting that a single amino acid alteration at the C-terminus of Aβ results in drastic distribution changes. These results suggest that MALDI-IMS could be used as a standard approach in combination with clinical, genetic, and pathological observations in understanding the pathology of AD and CAA.

**Electronic supplementary material:**

The online version of this article (10.1186/s40478-017-0477-x) contains supplementary material, which is available to authorized users.

## Introduction

Precise molecular identification of pathological depositions accelerates the diagnosis and clarifies the pathogenesis of neurodegenerative disorders [10]. In Alzheimer’s disease (AD) brains, depositions of insoluble amyloid β (Aβ) are detected in senile plaques (SP) before disease onset [1–3, 22, 23]. In addition to SP in the brain, Aβ is also deposited in the walls of cerebral capillaries and arteries and causes cerebral amyloid angiopathy (CAA) [27, 30, 32]. Although Aβ1–42 is predominant in SP, other Aβ variants, including N-terminal or C-terminal truncated or modified Aβs, are also identified in affected AD brains [4, 6, 18]. Characterizing and visualizing the broad Aβ species is needed to understand the Aβ-production, −metabolism, and -deposition, and may help elucidate the pathogenesis of AD and CAA.

In classical AD neuropathology, immunohistochemistry has been used to determine the localization of Aβs in brain tissues. However, the reliability of the results highly depends on the performance of antibodies, and the method cannot distinguish different variants when several epitopes are used simultaneously. Therefore, unbiased mass spectrometry-based proteomic analysis is a valuable approach to characterize the variety of Aβ species in brain tissues [9, 25, 26]. The matrix-assisted laser desorption/ionization (MALDI) mass spectrometric method has revealed different Aβ isoforms in homogenized brain lysates with immuno-precipitated samples [15]. However, this approach failed to reveal the detailed distribution of Aβ in the brain, and again, is dependent on the affinity of antibodies used. In recent years, MALDI-imaging mass spectrometry (IMS) has emerged as a powerful tool for investigating the distribution of proteins and small molecules within biological systems, through the in-situ analysis of tissue sections [21]. Here, we adopt this technology on postmortem brain tissues to extend and create a comprehensive protein mapping.

To analyze human brain samples, a novel protocol using formic acid pretreatment of brain tissues and an advanced type mass spectrometry, that bears advantages in its rapidity, sensitivity, and reproducibility, was needed. With the current technical advancements, we have successfully visualized distinct depositions of N- and C-terminal variations of Aβ in pathological human autopsied brains. The current strategy provides new insights into understanding the neuropathology of AD and CAA in terms of Aβ metabolism.

## Materials and methods

### Patients and brain specimens

Human cortical specimens for IMS and IHC were obtained from the Brain Bank at Tokyo Metropolitan Institute of Gerontology. Brains were removed, processed, and stored at −80 °C within 8 h postmortem. Patients were placed in a cold (4 °C) room within 2 h after death. All brains registered at the brain bank came with written informed consents for their use in medical research from the patients or their families. Each brain specimen was taken from the occipital cortex of five AD patients and five age-matched controls. This study was approved by the ethics committee at Doshisha University and Tokyo Metropolitan Geriatric Hospital.

In the present study, the extent of Aβ deposition as shown by an Aβ monoclonal antibody (12B2, 1:50 dilution; IBL, Gunma, Japan), was defined by Braak SP (amyloid) stages [2, 3]. At stage O, there are almost no senile plaques throughout the isocortex. At stage A, low densities of Aβ deposits are detected in the isocortex, particularly in the basal portions of the frontal, temporal, and occipital lobes. Furthermore, some plaques are found in the presubiculum and Pre-β and Pre-γ layers of the entorhinal complex. Stage B shows an increase in Aβ deposits in almost all isocortical association areas, and only the primary sensory areas and primary motor field remain practically devoid of deposits. There are mild amounts of deposits in the hippocampal formation, and Aβ deposits may be found in the entorhinal cortex. At stage C, virtually all the isocortical areas are affected, whereas deposits in the hippocampal formation display the same pattern as that of stage B. AD brains are invariable at stage C.

### Imaging mass spectrometry

Frozen tissue sections were cut on a cryostat (CM1950, Leica Microsystems, Wetzlar, Germany) at a 10 μm thickness using indium-tin-oxide–coated glass slides (Bruker Daltonics, Bremen, Germany). Prior to washing, stored samples were placed in a vacuum chamber to dry. To remove endogenous lipids and inorganic salts, dried samples were immersed in 70% ethanol for 30 s, pure ethanol for 30 s, Carnoy’s solution for 3 min, pure ethanol for 30 s, 0.1% TFA for 1 min, and pure ethanol for 30 s. Prior to matrix coating, treated with a formic acid vapor. Sinapinic acid was used as a matrix. For mass spectrometric measurements, tissue areas were defined using the FlexControl 3.8 and FlexImaging 5.0 software packages (both Bruker Daltonics). Spectra were acquired using the rapifleX MALDI Tissuetyper (Bruker Daltonics) in positive linear mode, where ions were detected in a mass range of *m/z* 2000 to 20,000, with spatial resolution of 20 and 100 μm, respectively. A ready-made protein standard was used for spectra calibration (Bruker Daltonics). Visualization and statistical analysis were completed using FlexImaging and SciLS Lab 2016a (SCiLS, Bremen, Germany).

### Immunohistochemistry

Fresh frozen sections were post-fixed using 4% paraformaldehyde (PFA) in phosphate buffered saline (PBS). After a brief wash in PBS, sections were stained by Sudan Black B for elimination of the autofluorescence due to lipofuscin [12]. Following immersion in 10% goat serum in PBS and 0.1% Tween20 (PBS-T) for 60 min, the sections were incubated with primary antibodies diluted in 1% BSA-PBS-T for 18 h at room temperature. Sections were then rinsed with PBS-T, and bound antibodies were visualized with secondary antibodies conjugated with Alexa dyes (Life Technology). The specimens were visualized by confocal-laser-scanning-microscope (LSM 700; Carl Zeiss Inc.). Whenever necessary, bound antibodies were labeled by biotinylated anti-mouse or anti-rabbit IgG antibodies (Vector Laboratories, Inc., Burlingame, CA), followed by avidin and biotinylated HRP complex (Vectastain Elite ABC kit; Vector Laboratories, Inc.). Bound HRP was developed with 3,3-diaminobenzidine (DAB) in the presence of hydrogen peroxide.

### Developing and characterizing anti-Aβ41 polyclonal antibody

The antigen peptide corresponding to Aβ37–41 amino acid residues, GGVVI, conjugated to thyroglobulin was immunized into rabbits four times at 2-week intervals. Subsequently, the serum was collected and purified with Aβ41 affinity chromatography. To confirm the purified antibodies’ specificity, synthetic Aβ1–40, Aβ1–41, and Aβ1–42 were run on a 12% Tris-tricine gel [7, 8] followed by western blotting. All Aβs were detected with 82E1 (IBL, Japan), while Aβ41 was detected with purified polyclonal antibody.

### Aβ aggregation test with Thioflavin T assay

Aβ was dissolved in hexafluoro isopropanol (HFIP) to 1.8 mg/ml. Each of the 230 μM Aβ samples were completely dried up. Samples were then dissolved in 50 mM NaPO_4_ (pH 7.4) and 0.25 mM NaOH. An equal volume of 56 mM thioflavin T was then added to each Aβ sample. Fifty microliters of the mixture was then added into the black wall plate individually and incubated at 37 °C with preventive light cover. The final Aβ concentration was 115 μM in each well. During sample incubation, thioflavin T intensity was measured for 24 h, at an excitation wavelength of 465 nm and emission wavelength of 535 nm.

## Results

### Depositions of Aβ1–40 and Aβ1–42 visualized with MALDI-IMS and IHC

To characterize the broad range of deposited and accumulated Aβ species in postmortem brain tissues, we adopted MALDI-IMS technology combined with formic acid pretreatment of brain tissues. Samples were obtained from sporadic AD patients with CAA (*n* = 5; mean age = 83.2 y) and SP free aged subjects (SP O) brains (n = 5; mean age = 77.2 y) as shown in Table 1.

**Table 1:** Clinical and pathological data of AD with CAA cases and aged SP O subjects

Subject No.	Gender	Age at death	Braak	BraakSP	CAA
1	M	83	5	C	0.5
2	M	88	5	C	1
3	M	84	5	C	2
4	M	78	6	C	1
5	M	83	5	C	1
6	M	84	1	O	0
7	M	78	1	O	0
8	M	70	1	O	0
9	M	73	1	O	0
10	M	81	1	O	0

**Fig. 1:**
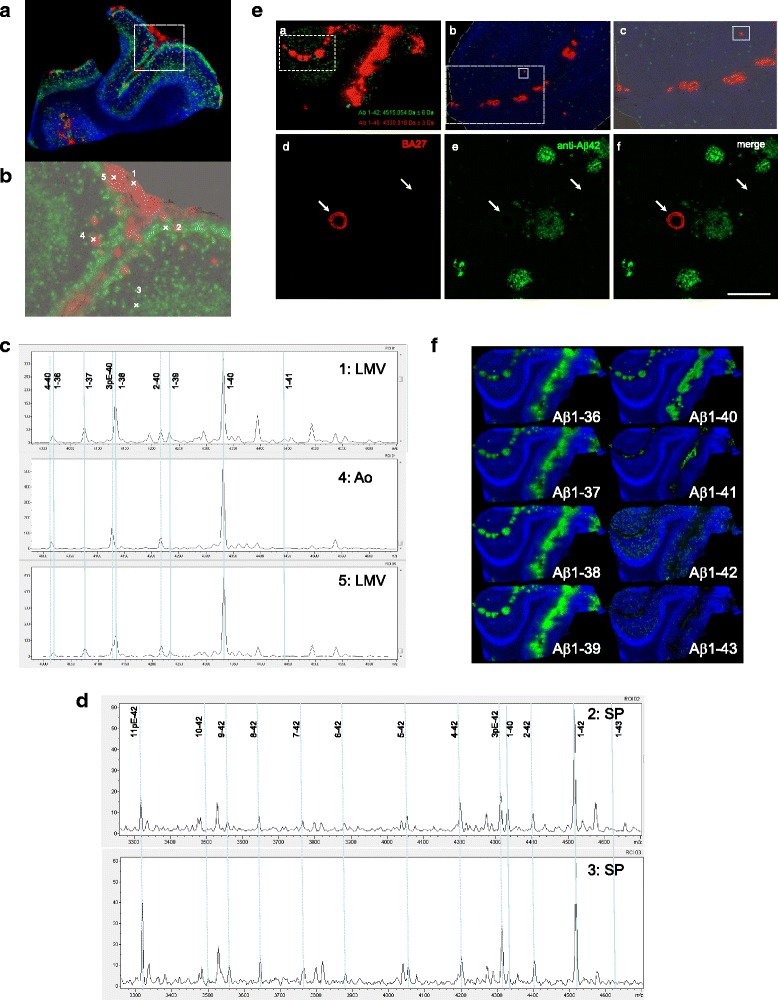
MALDI-IMS for frozen AD brain sections. **A**: Aβ1–40 deposits in the leptomeningeal blood vessels and arterioles (red) and Aβ1–42 deposits in cerebral parenchyma (green). The *m/z* 4939.9 was used to detect the tissue structure and shows an unknown biomolecule (blue). **B**: Optical density for MALDI-IMS. This figure is a magnification of the region within the dotted square in Fig. 1A. Aβ1–40 is deposited in leptomeningeal blood vessels (1 and 5) and arterioles (4) shown in red. Aβ1–42 is deposited in cerebral parenchyma as senile plaques (2 and 3) shown in green. **C**: MALDI Mass spectrum in leptomeningeal blood vessels (LMV), arterioles (Ao), and senile plaque (SP) of Fig. 1B. Aβ1–40 and N-terminal truncated Aβx-40 are located in Ao, while Aβ1–36 to Aβ1–41 are in LMV. Aβ1–42, Aβ1–43, and N-terminal truncated Aβx-42 are preferentially located in SP. **D**: MALDI-IMS and IHC of various C-terminal truncated Aβ peptides in AD with severe CAA. (**a**) MALDI-IMS 100 μm resolution imaging for Aβ1–40 (red) and Aβ 1–42 (green). (**b**) Highlight 20 μm resolution leptomeningeal blood vessels and cortex imaging in dotted square (**a**). (**c**) Highlight of an arteriole in solid square (**b**). Adjacent sections of the occipital cortex from AD brains were immunostained and focused on arteriole and cerebral parenchyma (**c**) using antibodies against Aβ40 (**d**: BA27) or Aβ42 (**e**: anti-Aβ42 polyclonal) and merged view (**f**). Both analyses demonstrated that Aβ40 is preferentially deposited in leptomeningeal blood vessels and arterioles in the subarachnoid space and the cerebral parenchyma forming CAA. In contrast, Aβ42 is mainly deposited in SP. IHC analysis also demonstrated the differential distribution of Aβ40 and Aβ42, which were CAA dominant and SP dominant deposition, respectively. Solid rectangles indicate the area illustrated in the panel. Scale bars = 100 μm. **E**: MALDI-IMS of various C-terminal truncated Aβ peptides in AD with severe CAA (NO. 3). Aβ1–36 to Aβ1–41 are preferentially deposited in leptomeningeal blood vessels, while Aβ1–42 and Aβ1–43 are deposited in the cerebral parenchyma as senile plaques

In case No. 4, with the most advanced Braak stage [2, 3], MALDI-IMS analysis identified distinct deposits of Aβ1–40 and Aβ1–42 in the AD brain tissue (Fig. 1A, B). The Aβ1–40 was distributed predominantly in the leptomeningeal vessels of the subarachnoid space and arterioles in the cerebral parenchyma. However, Aβ1–42 formed SP in the cerebral parenchyma, and was mostly deposited in the pyramidal cell layer and subpial molecular layer (Fig. 1A, B). Subpial depositions of Aβ1–42 are seen lining the subarachnoid space, but each MALDI-IMS signal pattern was more dispersed compared with pyramidal layer depositions due to the 100 μm pitch resolution. As shown in detail in Fig. 1C, the mass spectrum from leptomeningeal blood vessels in the subarachnoid space, arterioles, and cerebral parenchyma displayed different mass numbers corresponding to each Aβ ion. Aβ1–40 was detected not only in the blood vessels, but also along with Aβ1–42 in the cerebral parenchyma (Fig. 1C). The MALDI-IMS signal of Aβ1–40 from CAA is stronger than that of SP in the cerebral parenchyma (Fig. 1A, B). In normal brains, MALDI-IMS detected Aβ distribution as weak dot-like patterns (Additional file 1: Figure S1). Different distributions of Aβ40 and Aβ42 were also demonstrated by IHC using serial frozen tissue sections. The anti-Aβ40 antibody preferentially labeled CAA, which is in clear contrast to the distribution of Aβ42 in the cerebral parenchyma (Fig. 1D). It should be noted that these findings are further confirmed when the bound antibodies were visualized by the avidin-biotin-complex-DAB detection method (Additional file 1: Figure S2).

### Deposition of full-length Aβs visualized in human brain with MALDI-IMS

The CAA phenotypes of case No. 3 were the most advanced amongst subjects of this study. Interestingly, Aβ1–42 deposition in the subpial molecular layer was less prominent than in case No. 4 (Fig. 1A, E and Additional file 1: Figure S3). Furthermore, Aβ1–36 to Aβ1–41 were preferentially deposited in the leptomeningeal vascular areas, while both Aβ1–42 and Aβ1–43 were distributed in the cerebral parenchyma of the occipital cortex (Fig. 1E). This is the first study which detected Aβ1–41 in human brain tissue. We have hypothesized that one single amino acid alteration at the C-terminus of Aβ results in drastic changes in Aβs distribution. The spatial resolution used with MALDI-IMS is a key parameter and must be chosen carefully because high-resolution imaging often results in decreased sensitivity. In Fig. 1A and E, MALDI-IMS with 100 μm pitch resolution was used, and we obtained an overall distribution profile in a relatively wide area. To portray fine tissue structures, such as vessels, high-resolution MALDI imaging (20 μm) was performed. The MALDI-IMS clearly demonstrates that Aβ1–36 to Aβ1–41 are distributed in the leptomeningeal vessels and arteriole walls and are quite different from the SP distribution of Aβ1–42 and Aβ1–43 (Additional file 1: Figure S4). These results are in line with recent IHC findings citing that not only Aβ1–40, but also Aβ37 to Aβ39, are deposited in the leptomeningeal blood vessel walls [17].

### Deposition of N-terminal truncated/modified Aβ40 and Aβ42

**Fig. 2:**
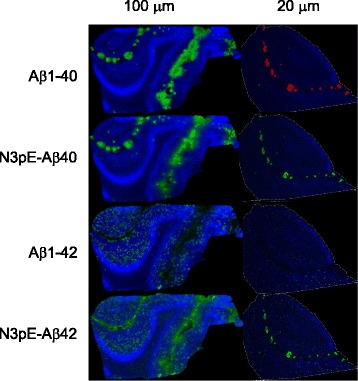
Aβ1–40/42 and N3pE-Aβ40/42 were detected at a 100 μm and 20 μm resolution IMS, respectively. Aβ1–40 and N3pE-Aβ40 preferentially deposited in leptomeningeal blood vessels and arterioles, while Aβ1–42 and N3pE-Aβ42 deposited in the cerebral parenchyma

A variety of different N-truncated Aβ peptides have been identified starting with amino residue Ala-2, N3pE, Phe-4, Arg-5, Asp-7, Ser-8, Gly-9, Tyr-10, and N11pE [15, 24]. In this study, the mass spectrometric profile of SP and CAA gave rise to serial Aβs like N3pE-Aβ and N11pE-Aβ in addition to many N-terminally truncated forms of Aβs (Fig. 1C). As shown here, MALDI-IMS distinguished between N-terminal truncated Aβ40 and Aβ42 with individual localization profiles in the brain tissues (Fig. 2). MALDI-IMS demonstrated that N3pE-Aβ40 is deposited in the leptomeningeal vessels and arterioles similarly to Aβ1–40, but was not present in SP. The *m/z* 4126.5 for N3pE-Aβ40 and 4132.5 for Aβ1–38 showed close to the mass number respectively (Fig. 1C). However, a detailed analysis with MALDI-IMS demonstrated that the *m/z* 4126.5 for N3pE-Aβ40 was distributed both in leptomeningeal blood vessels and arterioles, while the *m/z* 4132.5 for Aβ1–38 was detected only in leptomeningeal blood vessels (Fig. 1C). The mass number of N3pE-Aβ42 was detected not only in SP, but also in leptomeningeal blood vessels with MALDI-IMS (Fig. 2 and Additional file 1: Figures S5 and S6). Furthermore, MALDI-IMS obtained the detailed distributions of both Aβx-40 and Aβx-42 (x = 2, N3pE, 4, 5, 6, 7, 8, 9, 10, and 11pE) in AD accompanied with CAA brains (Additional file 1: Figures S5 and S6). These N-truncated Aβ species were largely similar in their distribution to the full-length Aβ1–40 and Aβ1–42. As shown here, MALDI-IMS can individually track the whole distribution of complex molecules having multiple modifications, an advantage over conventional IHC. Not only does C-terminus truncation cause different distribution patterns than N-terminus truncation, (Fig. 1A, B), it is plausible that the structures of its C-terminus structure predefine the dynamics of Aβs rather than those of the N-terminal end. It must be noted that the N3pE-Aβ42 may particularly behave independently from Aβ1–42. Furthermore, N11pE-Aβ42 was also detected in SP with MALDI-IMS as a relatively major peak (Fig. 1C and Additional file 1: Figures S5 and S6). This pyroglutamate modified Aβ has only been seen in SP [15, 24], while N11pE-Aβ40 was detected in leptomeningeal vessel walls, but not in SP (Additional file 1: Figures S5 and S6).

### Deposition of Aβ1–41 in contrast with Aβ1–40 and Aβ1–42

**Fig. 3:**
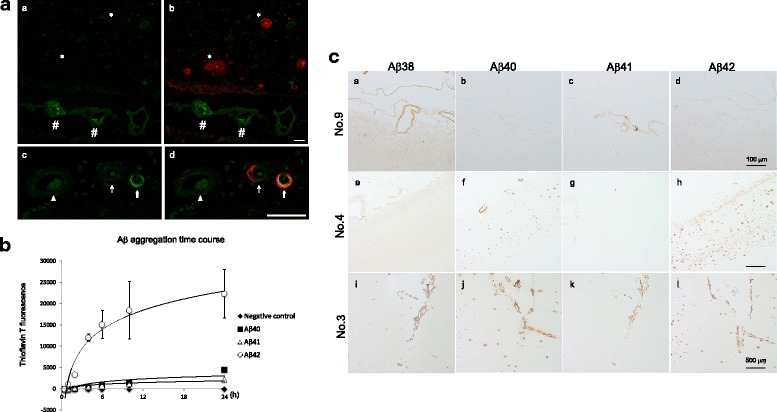
**A**: Aβ41 is deposited in leptomeningeal blood vessels. Frozen sections from an AD brain were subjected to the immunostaining using antibodies against Aβ41 (green in **a** − **d**), Aβ42 (red in **b**), and Aβ40 (red in **d**). Double immunostaining against both Aβ41 and Aβ42 demonstrated that the anti-Aβ41 antibody labeled the arterioles (#) in the subarachnoid space, but not the senile plaques (*) in the parenchyma (**a** and **b**). Double staining against Aβ41 and Aβ40 is shown. Three different stages of amyloid angiopathies, with weak or no Aβ41 deposition (arrowhead), modest Aβ41 deposition (thin arrow), and with severe Aβ41 deposition (thick arrow) are shown (**c** and **d**). Aβ41 was found in the amyloid angiopathy with severe Aβ40 deposition. Contrary to the Aβ40, which deposited in the periphery of adventitia, Aβ41 seemed to be localized in the smooth muscles layer of blood vessels. Scale bars = 50 μm. **B**: Time course of in vitro Aβ aggregation. Each synthetic Aβ incubated and measured thioflavin T fluorescence. Aβ1–42 aggregates immediately compared to the other variants. Aβ1–40 and Aβ1–41 were similar and showed little aggregation characteristic for 24 h. **C**: IHC for Aβs accumulation in the occipital cortex sections. Aβ38 and Aβ41 deposited in the leptomeningeal blood vessels in aged SP free brain (NO.9) (**a**, **c**). Aβs38, 40, and 41 also deposited in AD (NO.4) and CAA brains (NO.3), but Aβ40 had little deposits in the cortex, including arterioles (**e** − **g**, **i** − **k**). Amount of Aβ42 deposited in subpial granular cell layers and cortex (**h**, **l**). Scale bars = 100 μm (**a** − **d**) and 500 μm (**e** − **l**)

In Fig. 1C and E, we demonstrated the presence of Aβ1–41 peptide in leptomeningeal vessels similar to the full length Aβ species, Aβ1–36 to Aβ1–40. However, Aβ1–42 was predominantly deposited within the cerebral parenchyma. Aβ1–41 and Aβ1–42 showed clear differences in deposition patterns. To characterize Aβ1–41 in the brain, we have generated antibodies against Aβ41, as shown in Additional file 1: Figure S7. The IHC using anti-Aβ41 IgG labeled vessels with CAA as Aβ40, but was not in present in SP and showed a clear contrast to the antibodies against Aβ42 (Fig. 3A). To further test the hypothesis that such a drastic alteration of Aβ peptides distribution depends on its self-aggregation ability, we examined the in vitro aggregation characteristics of Aβ1–40, Aβ1–41, and Aβ1–42 using thioflavin T. Synthetic Aβ1–42 peptide aggregated immediately, while both synthetic Aβ1–40 and Aβ1–41 peptides aggregated slowly during a 24 h incubation (Fig. 3B). Thus, a C-terminal structure alteration from Aβ41 to Aβ42 is sufficient to alter its self-aggregation ability, and this structural difference may lead to SP formation in the cerebral parenchyma.

### Aβ38 and Aβ41 deposit in vessels prior to AD onset

We analyzed the accumulation of Aβ41 in contrast to Aβ38, Aβ40, and Aβ42 in the brain at various stages of SP. Surprisingly, Aβ38 and Aβ41 accumulated in the leptomeningeal blood vessels in the aged SP O subject brains as punctate (Fig. 3C). Furthermore, Aβ38 and Aβ41 were also detected in CAA in the brains of all cases having SP. Among the cases which had SP analyzed here, Aβ40 and Aβ42 were deposited in both SP and CAAs (Additional file 1: Table S1). In contrast to the Aβ40- and Aβ42-specific antibodies, the antibodies specific for Aβ38 and Aβ41 labeled only vessels regardless of the pathology. Therefore, it is presumed that Aβ38 and Aβ41 accumulate in vessels before the formation of the SP. Although we cannot confirm whether Aβ38 and Aβ41 positive vessels develop CAA, these Aβ species may be generated and accumulate into cerebral vessel walls in pre-pathological conditions.

## Discussion

Here we report a detailed study of Aβ distribution and its isoforms in brains with AD, CAA, and age-matched controls using MALDI-IMS and IHC. The current technical advantages of MALDI-IMS allowed us to unveil the distribution of various Aβ species within the same sections of human autopsied brains without specific probes. Furthermore, high resolution (20 μm) imaging of the brain of a subject with AD and severe CAA clearly demonstrates that Aβ1–36 to Aβ1–41 deposit into leptomeningeal vessel walls, while Aβ1–42 and Aβ1–43 aggregate in the cerebral parenchyma as SP. It is worth noting that MALDI-IMS detected Aβ deposition as an even dot-like pattern in normal control brains (Additional file 1: Figure S1). Considering that characterization of deposited Aβ by IMS must be in good agreement with IHC, both IMS and IHC were used and equally contributed to distinguishing Aβ deposits by their location, protein contents, and their morphology.

Multiple pathways of β-carboxyl-terminal fragment (β-CTF) processing by stepwise γ-secretase cleavage have been previously proposed [11, 16, 29]. In this model, Aβ1–41 is thought to exist in human AD brains, albeit as a minority. In the current study, we have succeeded in detecting the existence of Aβ1–41 in human brains by IMS and IHC for the first time. According to the Aβ processing model, Aβ1–38 is derived from Aβ1–45 via Aβ1–42, while Aβ1–41 is derived from Aβ1–45 by γ-secretase stepwise cleavage [11, 16, 29]. Of note, γ-secretase generates easily modifiable full-length Aβ1–36 to Aβ1–41 in the cerebral parenchyma and interstitial fluid (ISF) as a physiological step. Considering that γ-secretase activity is modulated in AD brains [8], and Aβ38 and Aβ41 have been detected in aged control brains (Fig. 3A, Additional file 1: Table S1), both modulation of γ-secretase activity and failure of Aβ drainage could be at cause for Aβ accumulation/deposition and CAA in AD brains [33].

An important finding of this study was that Aβ41 was associated with the smooth muscle of arteries, whereas Aβ40 was mostly in the adventitia. It is worth noting that Aβ41 appears to be constrained within the intramural periarterial drainage pathways whereas Aβ40 appears to have travelled radially across from the smooth muscle basement membranes to the pial-glial basement membranes (Fig. 3C, Additional file 1: Table S1). Previous studies showed that Aβ is eliminated from ISF through vascular basement membrane to lymph node and carotid artery in the neck, this system is known as the glymphatic system [14, 30, 32]. The Aβ elimination process is forced along the basement membrane of arterial walls by pulse wave [20], which is thought to be slowed in AD brains with aging [5, 31].

MALDI-IMS can individually track the whole distribution of complex molecules having multiple modifications, an advantage over conventional IHC. It is well known that N-terminal truncated Aβ peptides are abundant in brains of patients diagnosed with sporadic and familial AD [15, 24]. N3pE-Aβ42, more so than Aβ1–42, has been found as a major component of SP [4, 18]. However, antibodies against the N-terminus of N3pE-Aβs cannot distinguish both N3pE-Aβ40 and N3pE-Aβ42. Together with the views of a clear contrast of the distribution dependent on C-terminal truncation, it is plausible that the structures of its C-terminal end predefine the dynamics of Aβs rather than those of the N-terminal end. It must be noted that the N3pE-Aβ42 may particularly behave independently from Aβ1–42. Therefore, abundance of N-terminal truncated Aβx-42 (x = 2, N3pE, 4, 5, 6, 7, 8, 9, 10, and N11pE) and Aβ1–42 could form the aggregate core in SP. This explains how C-terminally truncated forms of Aβs are more soluble and likely become entrapped more distally in their drainage pathway. In contrast, the more fibrillogenic forms of Aβs are more prone to aggregation in the extracellular spaces of the brain and may not reach the drainage pathways, particularly in APOE4 positive individuals rather than APOE2/3 [13, 19, 28, 34].

## Conclusions

MALDI-IMS characterized a broad range of Aβ species deposits in brains with AD and CAA. Distinct depositions of N3pE-Aβ40 and N3pE-Aβ42 were comparatively visualized with Aβ40 and Aβ42. Aβ1–41 was first identified in human brains with MALDI-IMS and was confirmed by IHC. The deposition profile of Aβ1–41 drastically differs from Aβ1–42 in its aggregation ability. Thus, C-terminus of Aβ structure determines the deposition /accumulation location in AD brains.

## Additional file

Additional file 1: Figure S1. MALDI-IMS for SP free control subjects. **Figure S2**. IHC for Aβ40 (BA27) or Aβ42 (BC05) in brain with AD and CAA. **Figure S3**. MALDI-IMS of AD brain (No.3) merged on optic density figure. **Figure S4**. High resolution (20 μm) figure of MALDI-IMS for various C-terminal truncated Aβ in AD with severe CAA.**Figure S5**. MALDI-IMS for various N-terminal truncated and modified Aβs in AD with moderate CAA. **Figure S6**. MALDI-IMS for various N-terminal truncated and modified Aβs in AD with severe CAA. **Figure S7**. Anti-Aβ41 antibody characterization. Supplementary **Table S1**. (PDF 1494 kb)
