# Supplementary material for: Distinct deposition of amyloid-β species in brains with Alzheimer’s disease pathology visualized with MALDI imaging mass spectrometry
Source: Acta Neuropathol Commun. 2017 Oct 16;5:73. doi: 10.1186/s40478-017-0477-x (PMC5641992; doi:10.1186/s40478-017-0477-x)
Supplement: Additional file 1: Figure S1. — MALDI-IMS for SP free control subjects. Figure S2. IHC for Aβ40 (BA27) or Aβ42 (BC05) in brain with AD and CAA. Figure S3. MALDI-IMS of AD brain (No.3) merged on optic density figure. Figure S4. High resolution (20 μm) figure of MALDI-IMS for various C-terminal truncated Aβ in AD with severe CAA.Figure S5. MALDI-IMS for various N-terminal truncated and modified Aβs in AD with moderate CAA. Figure S6. MALDI-IMS for various N-terminal truncated and modified Aβs in AD with severe CAA. Figure S7. Anti-Aβ41 antibody characterization. Supplementary Table S1. (PDF 1494 kb) [file 40478_2017_477_MOESM1_ESM.pdf]

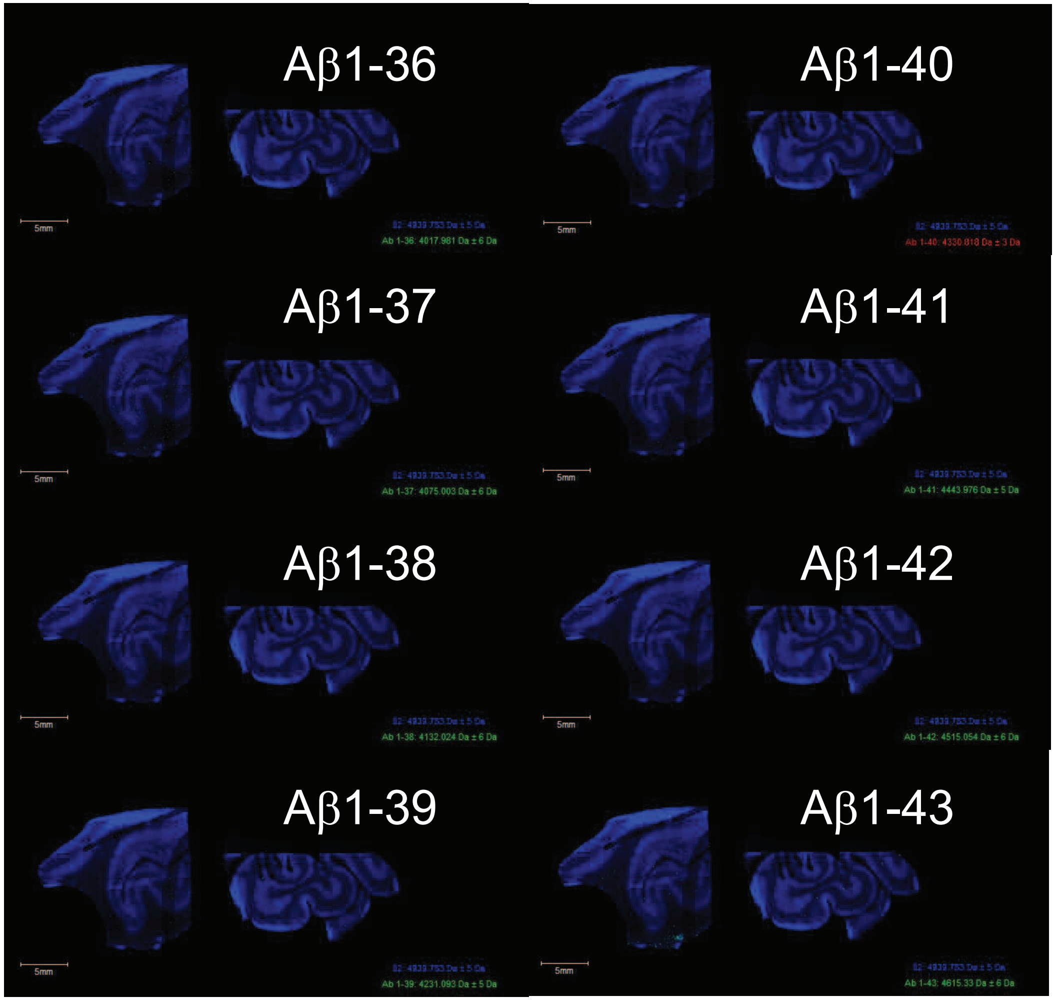

No.10, No.9

Supplementary figure S1

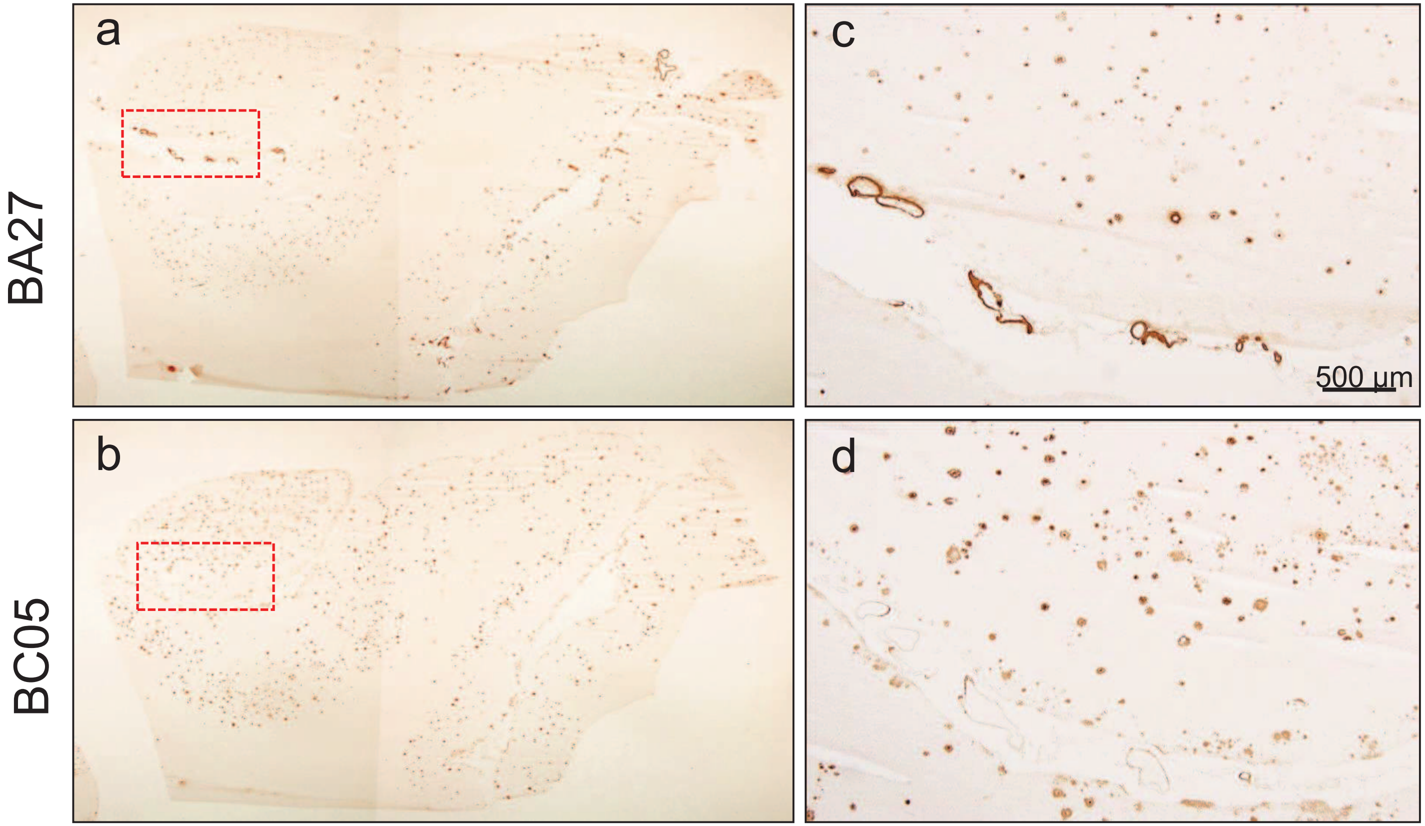

No.3

Supplementary figure S2

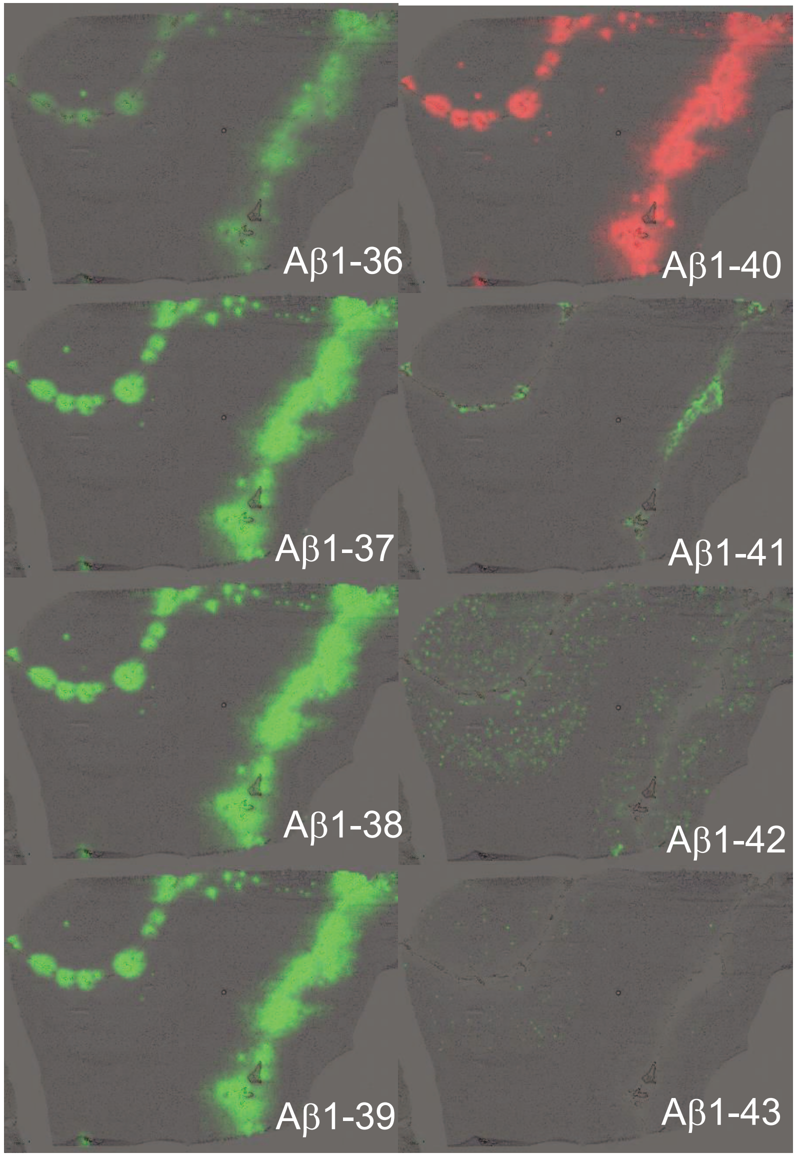

Supplementary figure S3

No.3

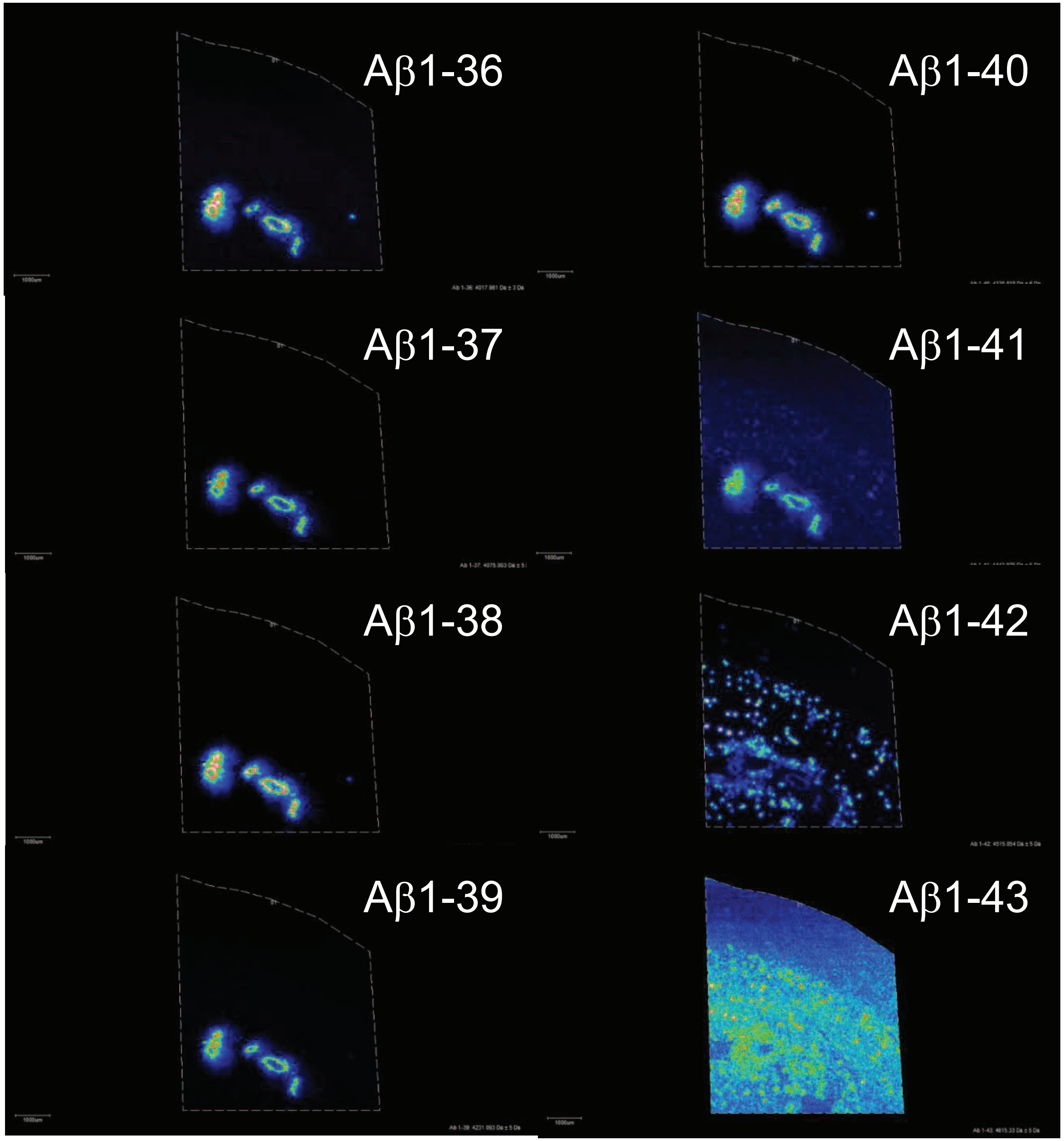

Supplementary figure S4

No.3

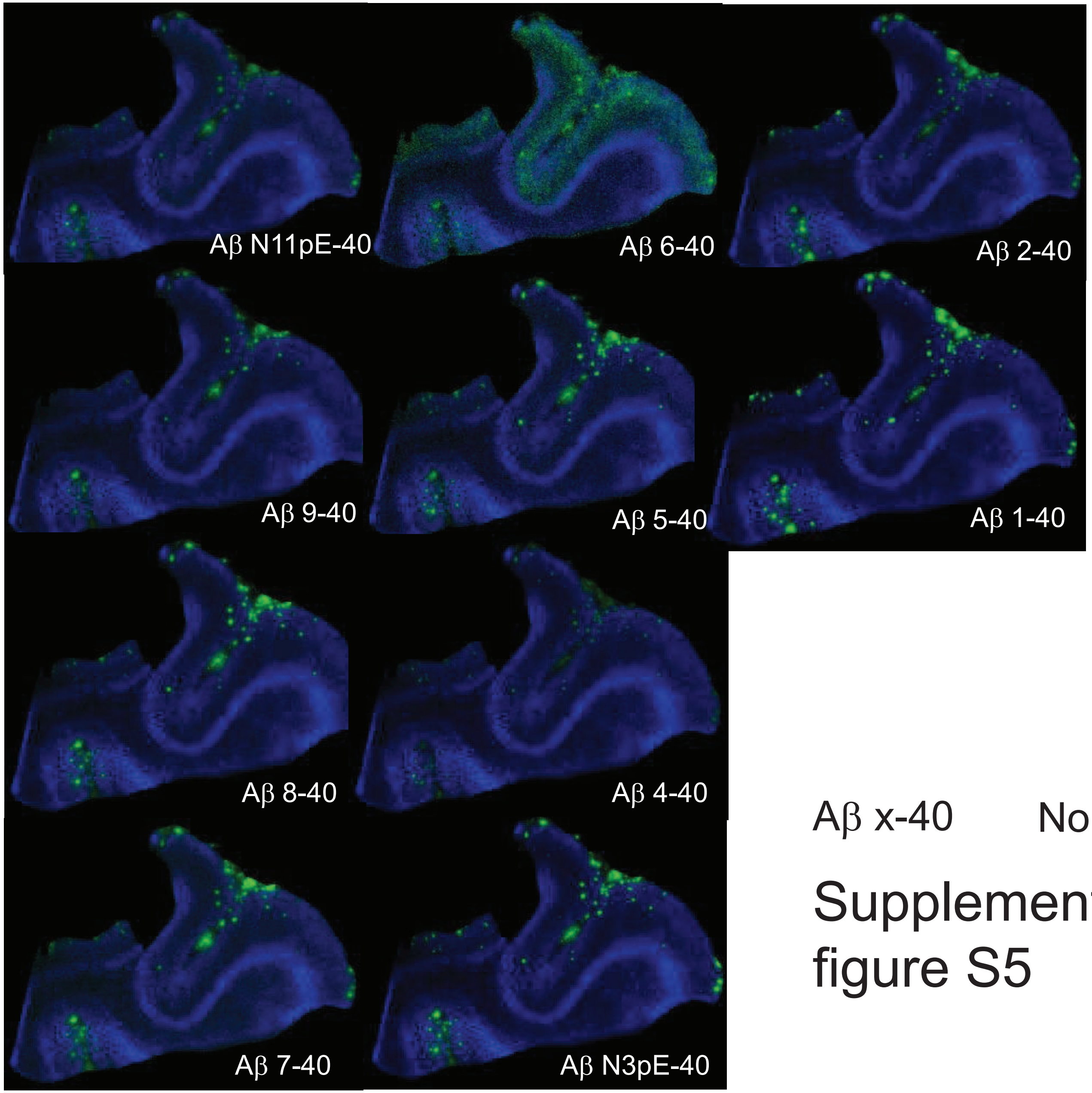

Aβ x-40 No.4

Supplementary figure S5

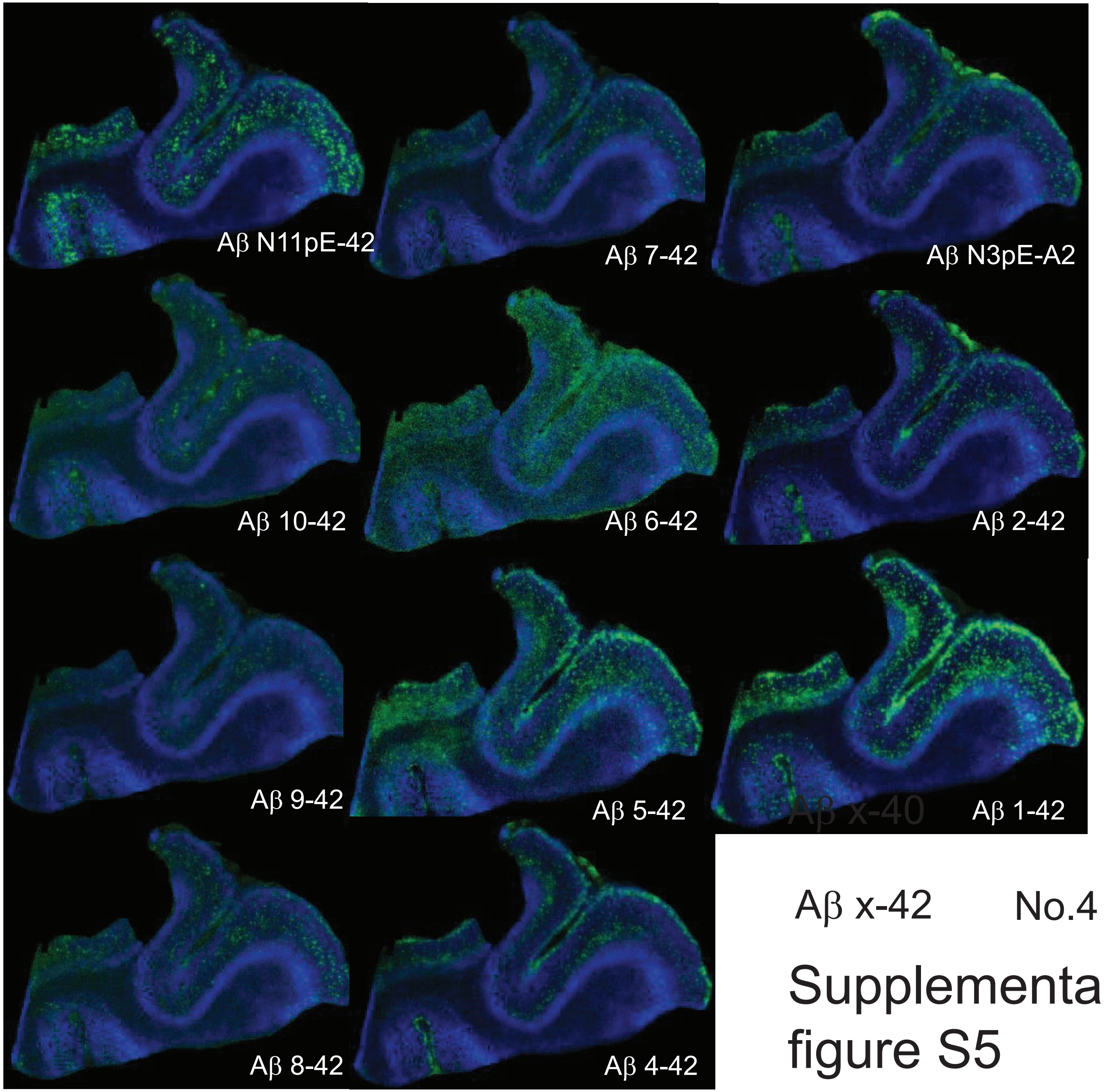

Aβ x-42 No.4

Supplementary figure S5

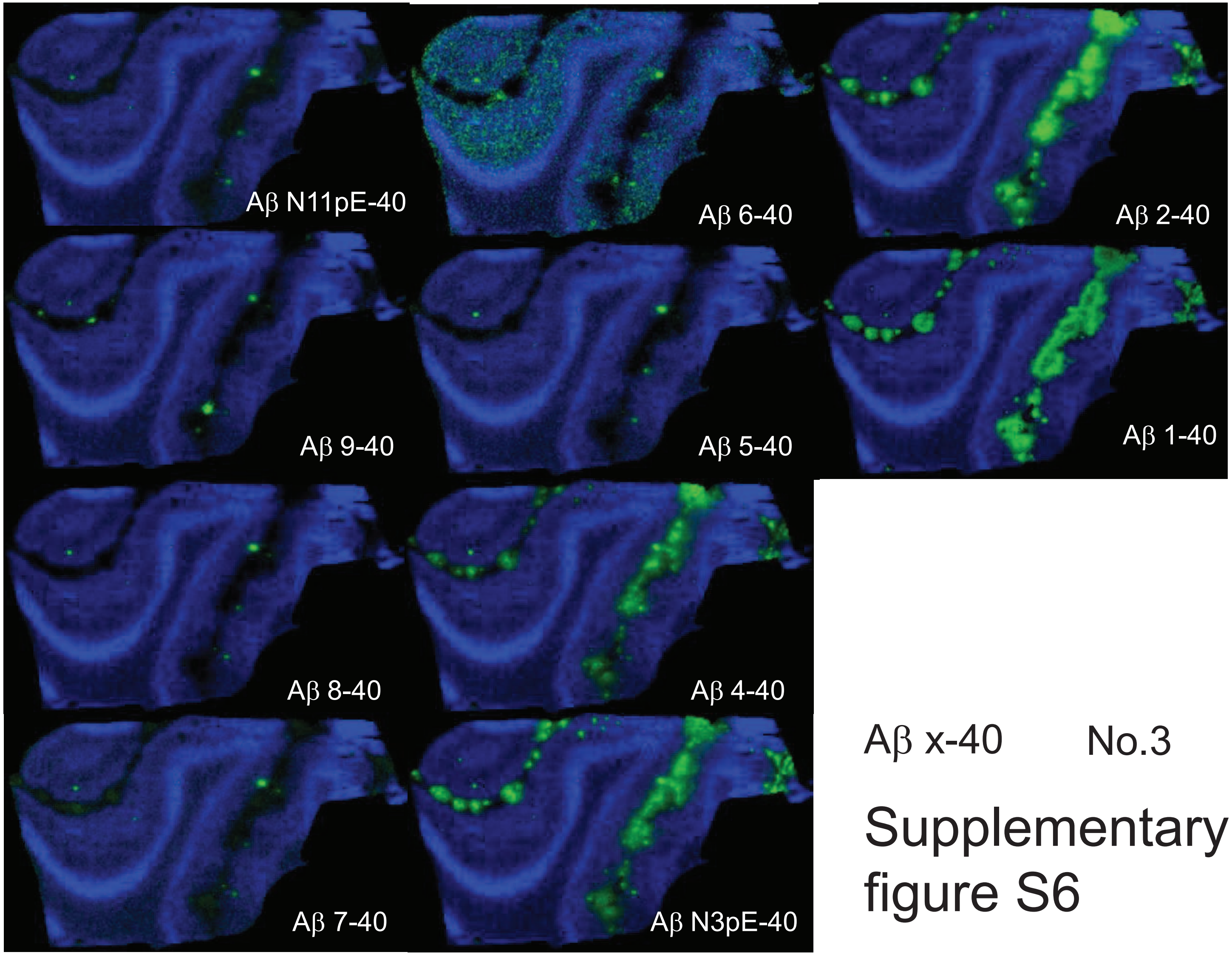

Aβ x-40 No.3

Supplementary figure S6

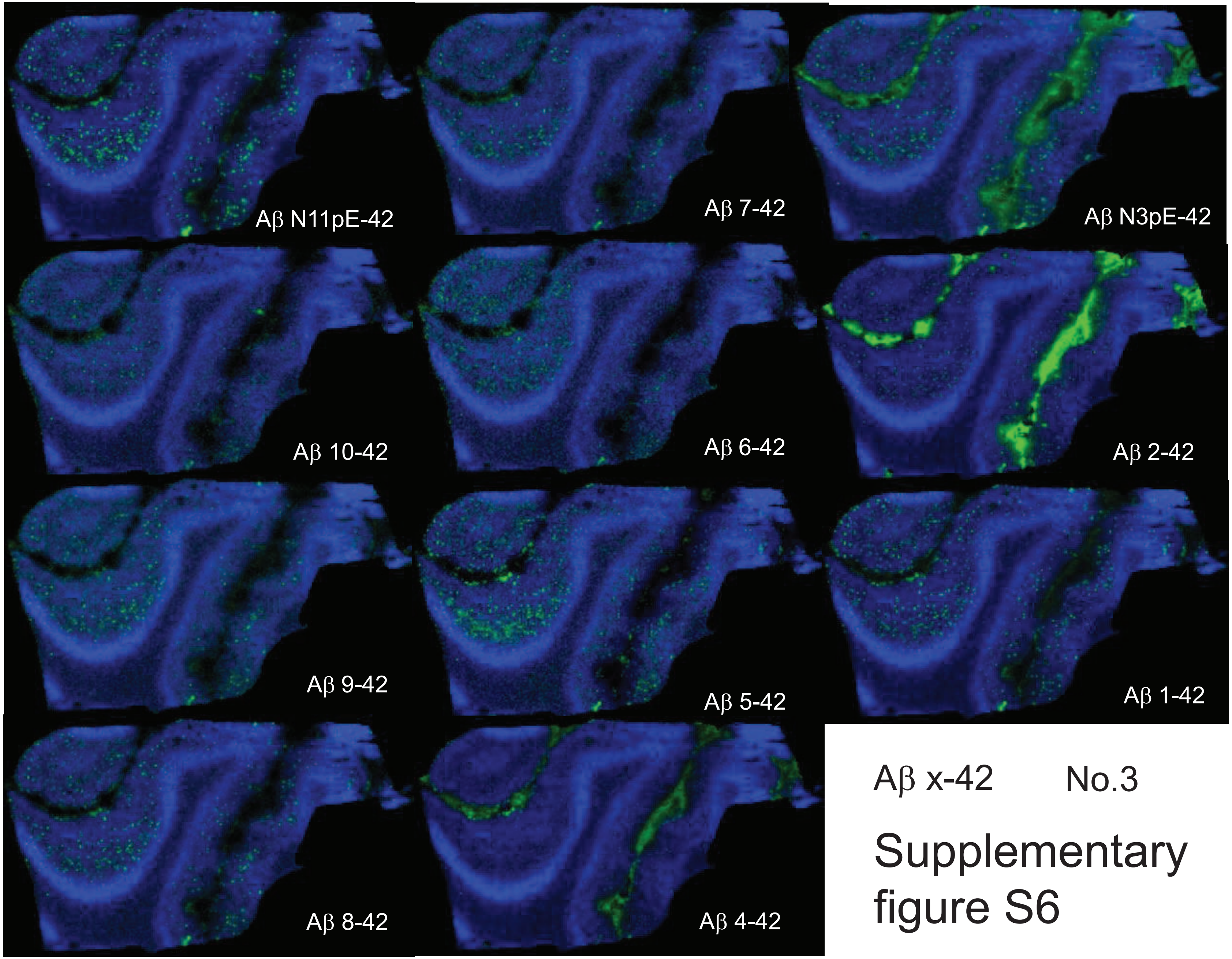

Aβ x-42 No.3

Supplementary figure S6

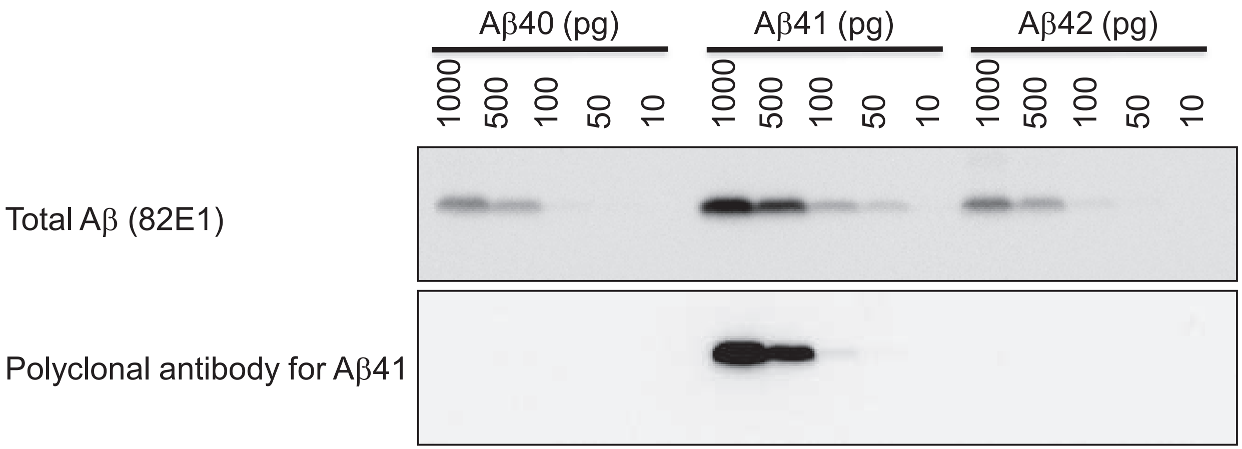

Supplementary figure S7

No.	Braak SP	CAA	Senile plaque				CAA in meninges				CAA in parenchyma			
			Aβ38	Aβ40	Aβ41	Aβ42	Aβ38	Aβ40	Aβ41	Aβ42	Aβ38	Aβ40	Aβ41	Aβ42
1	C	0.5	0	1	0	1	1	0	1	0	0	0	0	0
2	C	1	0	1	0	1	2	2	2	2	1	1	0	1
3	C	2	0	1	0	1	3	3	3	2	3	3	3	2
4	C	1	0	1	0	1	3	3	3	2	2	2	2	2
5	C	1	0	1	0	1	2	2	2	1	1	2	1	1
6	O	0	0	0	0	0	1	0	1	0	0	0	1	0
7	O	0	0	0	0	0	1	0	1	0	0	0	0	0
8	O	0	0	0	0	0	1	0	2	0	1	0	1	0
9	O	0	0	0	0	0	1	0	2	0	1	0	2	0
10	O	0	0	0	0	0	1	0	1	0	0	0	0	0

Supplementary table S1
